# Illustrated Scientific American

**Published:** 1862-01

**Authors:** 


### ILLUSTRATED SCIENTIFIC AMERICAN.

"We do not believe that even in this age of cheap publications any work can be more reasonable than the terms of the SCIENTIFIC AMERICAN at $2 per annum, with twenty-five per cent. discount for clubs of ten. It forms a yearly volume of 832 pages quarto, with an immense number of original engravings of patented machines, valuable inventions, and objects of scientific interest. There is not an industrial pursuit which does not receive a share of its attention. It contains official lists of patent claims, important statistics, practical recipes for useful domestic purposes, and has long stood, both in this country and Europe, as the highest authority in the mechanic arts and sciences. There is no publication more valuable to the farmer, the miller, the engineer, the iron founder, the mechanic, or the manufacturer. We have never opened a number without learning something we never knew before, and obtaining valuable information for the benefit of our readers. The Publishers, Messrs. MUNN & Co., of 37 Park Row, New York, have deserved the success which they have achieved. No one should visit that city without calling at their palatial establishment, which is a museum of inventive genius, collected from the entire world. If any of our friends away off in the country do not know this work, and will take our advice, they wlll mail $2 and become subscribers immediately, or by applying to the Publishers they can obtain a specimen copy gratis, which will be sure to confirm the truth of our recommendation."—*Louisville Jouonal.*

We fully indorse the above, and would recommend our readers to take Prentice's advice, and subscribe for the paper. A new volume commences on the first of January, and it being a valuable work of reference, containing, as it does, the only official list of patent clalms published in the country, every number should be preserved. The paper is published every Saturday, by the well-known patent agents, Messrs. MUNN & Co., who have conducted the paper during the past sixteen years.

In addition to furnishing specimen copies of the paper gratis, the publishers will send a pamphlet of advice to inventors, free of charge.

Address, MUNN & CO., 37 Park Row, New York.

